# Agmatine Alleviates Epileptic Seizures and Hippocampal Neuronal Damage by Inhibiting Gasdermin D-Mediated Pyroptosis

**DOI:** 10.3389/fphar.2021.627557

**Published:** 2021-08-06

**Authors:** Xueying Li, Jiahe Lin, Yingjie Hua, Jiaoni Gong, Siqi Ding, Yanru Du, Xinshi Wang, Rongyuan Zheng, Huiqin Xu

**Affiliations:** Department of Neurology, the First Affiliated Hospital of Wenzhou Medical University, Wenzhou, China

**Keywords:** epilepsy, agmatine, pyroptosis, inflammation, GSDMD

## Abstract

**Background:** Epilepsy is a common neurological disease, and neuroinflammation is one of the main contributors to epileptogenesis. Pyroptosis is a type of pro-inflammatory cell death that is related to epilepsy. Agmatine, has anti-inflammatory properties and exerts neuroprotective effects against seizures. Our study investigated the effect of agmatine on the core pyroptosis protein GSDMD in the context of epilepsy.

**Methods:** A chronic epilepsy model and BV2 microglial cellular inflammation model were established by pentylenetetrazole (PTZ)-induced kindling or lipopolysaccharide (LPS) stimulation. H&E and Nissl staining were used to evaluate hippocampal neuronal damage. The expression of pyroptosis and inflammasome factors was examined by western blotting, quantitative real-time PCR, immunofluorescence and enzyme-linked immunosorbent assay (ELISA).

**Results:** Agmatine disrupted the kindling acquisition process, which decreased seizure scores and the incidence of full kindling and blocked hippocampal neuronal damage. In addition, agmatine increased BV2 microglial cell survival *in vitro* and alleviated seizures *in vivo* by suppressing the levels of PTZ-induced pyroptosis. Finally, the expression of TLR4, MYD88, phospho-IκBα, phospho-NF-κB and the NLRP3 inflammasome was significantly upregulated in LPS-induced BV2 microglial cells, while agmatine suppressed the expression of these proteins.

**Conclusions:** Our results indicate that agmatine affects epileptogenesis and exerts neuroprotective effects by inhibiting neuroinflammation, GSDMD activation, and pyroptosis. The inhibitory effect of agmatine on pyroptosis was mediated by the suppression of the TLR4/MYD88/NF-κB/NLRP3 inflammasome pathway. Therefore, agmatine may be a potential treatment option for epilepsy.

## Introduction

Epilepsy is a common chronic neurological disorder that is characterized by recurrent seizures caused by the abnormal discharge of neurons. Currently, there are more than 7 million epilepsy patients worldwide (Fiest et al., 2017). In 2015, there were approximately 9.84 million lifelong epilepsy patients in China (Song et al., 2017). Epilepsy brings great obstacles to patient lives, but the pathogenesis is not fully understood. There is accumulating evidence showing that inflammation plays an important role in the progression of epilepsy (Friedman and Dingledine, 2011; Vezzani et al., 2008). Damage to the brain, usually triggers a neuroinflammatory response, characterized by an increase in pro-inflammatory molecules, which mediates the recurrence of epilepsy (Musto et al., 2011; Musto et al., 2016). Therefore, anti-inflammatory factors may play a role in preventing epileptic seizures and neuroprotection.

Inflammation is a multiprotein process that involves cytoplasmic pattern recognition receptors. Some studies have shown that inflammation is involved in the pathophysiological processes of many central nervous system diseases, including Parkinson’s disease (Han et al., 2019), Alzheimer’s disease (Goldmann et al., 2013) and epilepsy (Magalhaes et al., 2018). In animal models of epilepsy, cerebral inflammasome levels are increased, and inhibiting the expression of inflammatory bodies may significantly reduce the loss of neurons and the severity of seizures (Meng et al., 2014; Rong et al., 2019). Recent studies have shown that sinomenine exerts anticonvulsant and neuroprotective effects in pentylenetetrazole (PTZ)-kindled rats by inhibiting inflammatory activation (Gao et al., 2018). Moreover, Tan and colleagues found that pro-inflammatory programmed cell death-known as pyroptosis plays a key role in the epileptogenic process (Tan et al., 2015). Pyroptosis, which was first identified by Brennan in 2001 (Cookson and Brennan, 2001), is divided into the classic pathway mediated by caspase-1 cleavage and the non-classic pathway mediated by caspase-4/5/11 cleavage (Shi et al., 2017). Emerging evidence has shown that the protein gasdermin D (GSDMD) is cleaved by caspase-1/4/5/11 to gasdermin D-N domains (GSDMD-N), which is the executor of pyroptotic cell death (Shi et al., 2015), releasing the inflammatory cytokines Interleukin-1β (IL-1β) and IL-18. However, the role of GSDMD and its related mechanism in epilepsy remain to be elucidated. We aimed to investigate the role of GSDMD in epilepsy and its mechanism.

Agmatine (AGM) has anti-inflammatory effects (McCurtain et al., 2019) and has been shown to be associated with a variety of neurological diseases, including Alzheimer's disease (Kotagale et al., 2020), ischaemic stroke (Kim et al., 2006), traumatic brain injury (Kim et al., 2015) and Parkinson’s disease (Matheus et al., 2012). Agmatine is an endogenous amine synthesized from the decarboxylation of L-arginine, which is mediated by the enzyme arginine decarboxylase. Agmatine is a novel neuromodulator and neurotransmitter that has been identified in many animal organs, especially in the brain (Raasch et al., 2001; Moretti et al., 2014). An extensive body of evidence has demonstrated that PTZ-induced seizures in rats can be alleviated by the application of agmatine (Feng et al., 2005; Bahremand et al., 2010). Our previous study also showed that a high dose of agmatine attenuated PTZ-induced chronic seizures in rats by exerting anticonvulsive effects (Xu et al., 2014). Additionally, agmatine also effectively inhibits the transcription factor NF-κB (Li et al., 2020). The present study was designed to explore the molecular targets of the anti-inflammatory and anti-pyroptotic effects of agmatine in epilepsy-associated signal transduction pathways in mice with PTZ-induced seizures and lipopolysaccharide (LPS)-induced microglial cells.

## Methods

### Animals

Thirty male C57L/B6 mice (6–8 weeks, 20–30 g) were obtained from the experimental animal centre of Wenzhou Medical University and housed under a 12-h light-dark cycle with food and water ad libitum. All experimental procedures were approved by the Ethics Committee of Wenzhou Medical University and were conducted in accordance with the National Institutes of Health Guide for the Care and Use of Animals.

### Chemical Kindling Model

Epileptic seizures were induced in the mice using PTZ as described previously (Tang et al., 2017). Mice were intraperitoneally injected with PTZ (35 mg/kg, Sigma, St. Louis, MO, United States) once every other day for a total of 14 injections (from day 1 to day 28). Agmatine (10 mg/kg. Sigma) was injected 30 min before the administration of PTZ (Payandemehr et al., 2013). At 1 h after PTZ injection, the behaviour of each mouse was observed. Seizure activity was scored using the criteria described by Racine (1972) (Racine, 1972). Mice with at least three consecutive stage 4 or 5 seizures were regarded as kindled. Seizure stages were classified as follows: stage 0, no response; stage 1, ear and facial twitching; stage 2, myoclonic jerks (MJs); stage 3, clonic forelimb convulsions; stage 4, generalized clonic seizures with turning to a side position; and stage 5, generalized tonic-clonic seizures (GTCSs) or death.

To investigate the neuroprotective effects of agmatine, the mice were randomly divided into three groups, with 10 mice in each group: 1) in the control group (saline-saline group), saline was used as a negative control; 2) in the model group (saline-PTZ group), the mice were injected with saline 30 min before PTZ injection; and 3) in the treatment group (agmatine-PTZ group), the mice were injected with agmatine 30 min before PTZ injection.

### Cell Lines and Culture Conditions

The mouse microglial cell line, BV2, was purchased from the German Collection of Microorganisms and cell cultures (DSMZ). The cells were cultured in Dulbecco’s modified Eagle’s medium (DMEM; Thermo Fisher Scientific, Waltham, MA, United States) containing 10% fetal bovine serum (FBS; Thermo Fisher Scientific) at 37°C in 5% CO_2_. Preliminary experiments were performed to determine the dose of agmatine. BV2 microglial cells were pretreated with 1, 10, 50, 100, 200 or 500 μM agmatine. The dose chosen for subsequent experiments was 100 μM agmatine.5 µM Bay-117082 (an NF-κB pathway inhibitor) (S2913; Selleck, Shanghai, China), or a Nucleotide oligomerization domain (NOD)-like receptor protein 3 (NLRP3) inflammasome inhibitor (10 µM, S3680; Selleck) was used to BV2 microglial cells for 1 h before LPS stimulation.

BV2 microglial cells were divided into four groups: the control group was administered sterile water; the AGM group was administered agmatine alone (100 μM); the LPS group was administered LPS (1 μg/ml) (Nam et al., 2018); and the LPS + AGM group was administered agmatine (100 μM) followed by LPS (1 μg/ml).

### Assessment of Cell Viability With CCK-8 Assay

A CCK-8 assay (C0038; Beyotime) was used to assess cell survival. BV2 microglial cells were seeded into 96-well plates at a density of 4*10^3^ per well with agmatine at different concentrations in the absence of FBS. The experiment was performed according to the manufacturer’s protocol. The kit contains a water-soluble tetrazolium salt (WST-8), which is the substrate for the mitochondrial succinate dehydrogenases that convert WST-8 into formazan (orange-coloured). The activities of these enzymes are proportional to the number of viable cells. BV2 microglial cells were seeded in 96-well plates and the control group, AGM group, LPS group, and LPS + AGM group were exposed to various culture conditions. We analysed the intensity of the colour development by measuring the absorbance at 450 nm in each well.

### Western Blotting

The protein levels were examined by immunoblotting. All mice were administered 1% pentobarbital sodium and decapitated, and the bilateral hippocampus was immediately collected. Hippocampal tissue samples (20–30 mg) were lysed, the samples were centrifuged at 12,000 g for 15 min, and then the supernatants were collected. Protein samples (20 μg) were separated by SDS-PAGE (10% separation gel) and transferred to a PVDF membrane (Merck and Co., Inc., Whitehouse Station, NJ, United States, Germany). The PVDF membranes were incubated with 5% nonfat milk for 1 h at room temperature (RT). Then, the PVDF membranes were incubated overnight at 4°C with the following primary antibodies: GSDMD (sc-393581, 1:200; Santa Cruz Biotechnology), NLRP3 (ab263899, 1:1,000; Abcam, Cambridge, United Kingdom), Caspase-1 (ab179515, 1:1,000; Abcam, Cambridge, United Kingdom), IL-1β (ab234437, 1:1,000; Abcam, Cambridge, United Kingdom), TLR4 (#14358, 1:1,000; Cell Signaling Technology), MYD88 (#4283, 1:1,000; Cell Signaling Technology), NF-κB (p65, #8242, 1:1,000; Cell Signaling Technology), phospho-NF-κB (p-p65, #3033, 1:1,000; Cell Signaling Technology), IκBα (#4814, 1:1,000; Cell Signaling Technology, Danvers, MA, United States), phospho-IκBα (*p*-IκBα, #9246, 1:1,000; Cell Signaling Technology), ASC/TMS1 (#67824, 1:1,000; Cell Signaling Technology), and GAPDH (#5174, 1:1,000; Cell Signaling Technology). The horseradish peroxidase (HRP)-conjugated goat anti-mouse (A0216, 1:2,000; Beyotime) and goat anti-rabbit (A0208, 1:2,000; Beyotime) secondary antibodies were added and incubated with the PVDF membranes for 1 h at RT after the membranes were washed three times. The ECL method (Bio-Rad) was used to detect the proteins. The proteins levels were normalized to GAPDH (#5174, 1:1,000; Cell Signaling Technology).

### RT-PCR

The mRNA expression in the hippocampus was determined by real-time quantitative RT-PCR. Total RNA was extracted using TRIzol reagent (Thermo Fisher Scientific). Total RNA was reverse-transcribed using the Revert Aid First Strand cDNA synthesis kit (#K1622; Thermo Fisher Scientific) and the synthesized cDNA was used for RT-PCR with SYBR (#RR037A; Takara Biotechnology, Dalian, China). PCR was performed on an ABI7500 platform. All results were normalized to GAPDH. The following specific primers were used:

GSDMD: forward primer: 5’-CCA​TCG​GCC​TTT​GAG​AAA​GTG-3’ and reverse primer: 5’-ACA​CAT​GAA​TAA​CGG​GGT​TTC​C-3’;

NLRP3: forward primer: 5‘-TGGATGGGTTT GCTGGGAT-3’ and reverse primer:5‘-CTGCGTGTAGCGACTGTTGAG-3’;

ASC: forward primer:5‘-CTG GAG​TCG​TAT​GGC​TTG​GAG-3’ and reverse primer: 5‘-CAA​AGT​GTC​CTG​TTC​TGG​CTG​TA-3’;

Caspase-1: forward primer: 5‘-TTG​AAA​GAC​AAG​CCC​AAG​GTG-3’ and reverse primer: 5‘-CTGGTGTTGAAGA GCAGAAAGC-3’;

IL-1β: forward primer: 5‘-ACC​TTC​CAG​GAT​GAG​GAC​ATG​A-3’ and reverse primer: 5‘-AAC​GTC​ACA​CAC​CAG​CAG​GTT​A-3’;

GAPDH: forward primer 5’-GAC​ATG​CCG​CCT​GGA​GAA​AC-3’ and reverse primer: 5’-AGC​CCA​GGA​TGC​CCT​TTA​GT-3’.

### ELISA

After the indicated treatments, the supernatants and the serum were collected. The level of IL-1β was measured by commercial ELISA kits (R&D Systems), according to the manufacturer’s protocol.

### Haematoxylin and Eosin (H&E) Staining

Hippocampal tissue from the mice was fixed in 4% paraformaldehyde, and then embedded in paraffin. The paraffinized brains were cut into 5 um-thick sections and stained with H&E. The hippocampal CA1 and CA3 areas were examined by a microscope (Olympus Corporation, Tokyo, Japan).

### Nissl Staining

Hippocampal tissues were embedded in paraffin, and 5 um-thick sections were stained with Nissl staining solution. After the sections were dehydrated in ethanol and washed with distilled water, the sections were cleared with xylene, sealed with neutral gum, and viewed under the microscope (Olympus Corporation, Tokyo, Japan).

### Immunofluorescence Analysis

Hippocampal tissue from the mice was fixed in 4% paraformaldehyde, and then embedded in paraffin. The paraffinized brains were cut into 5 um-thick sections. For cellular immunofluorescence, BV2 microglial cells were seeded into 6-well plates at a density of 0.25-1*10^6^ per well. BV2 microglia were plated in confocal dishes and treated with LPS (1 μg/ml) for 24 h with or without agmatine. The cells were fixed with 4% paraformaldehyde for 10 min. Next, the cells and tissue sections were permeabilized with 0.25% Triton X-100 in PBS for 10 min. The cells and tissue sections were washed 3 times with PBS before being blocked with 1% BSA for 30 min. After being rinsed with PBS, the tissue sections were incubated with anti-GSDMD (1:50) antibodies, and the cells were incubated with anti-P65 (1:50) antibodies at 4°C overnight. The primary antibodies were removed, and the plate was washed 3 times with PBS. The samples were then incubated with secondary antibodies for 1 h at RT. After being washed with PBS, the nuclei were stained with DAPI for cell localization.

### Statistical Analysis

Statistical analysis was performed by GraphPad Prism 7 (GraphPad, San Diego, CA, United States). The assumptions of normality were verified using the Shapiro-Wilks test and equal variance was checked using Levene’s test. Two-way analysis of variance (ANOVA) with repeated measures followed by Bonferroni or Dunnett’s T3 post hoc test was used to analyze data related to seizure stage. Student’s test was used to compare variables between two groups. One-way analysis of variance (ANOVA) was used to analyse differences among groups followed by LSD post hoc comparisons when appropriate. All data are presented as the mean ± standard deviation (SD). A value of *p*＜0.05 was considered statistically significant.

## Results

### Agmatine Alleviates Seizure Susceptibility in Mice With PTZ-Induced Kindling

**FIGURE 1:**
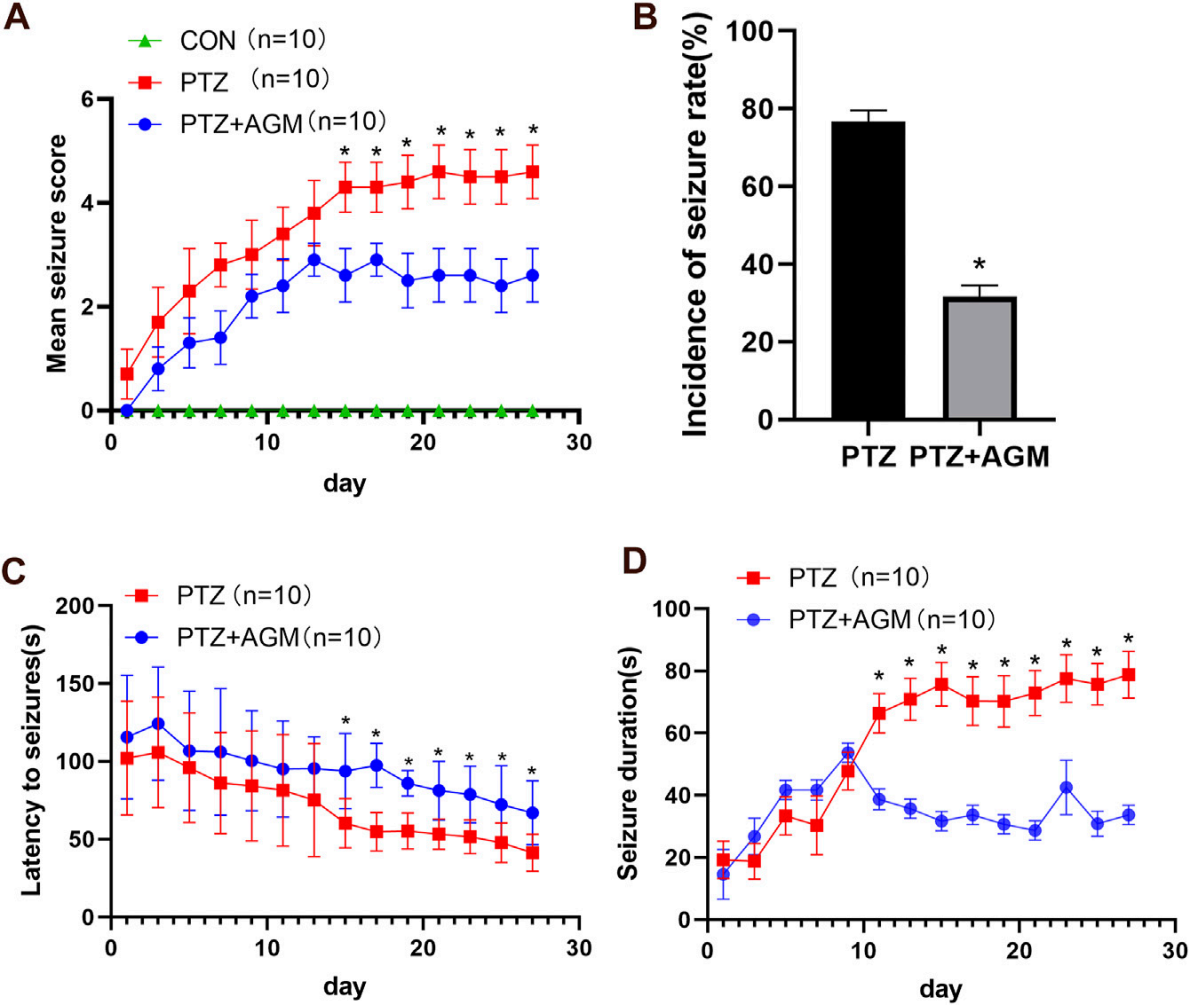
Effects of AGM on PTZ kindling-induced seizure. **(A)** Statistical result showing AGM decreased seizure score (two-way ANOVA with repeated measures followed by Bonferroni or Dunnett’s T3 post hoc test). **(B)** Statistical results showing AGM decreased the incidence of seizure rate (chi-square test). **(C)** Statistical results showing AGM increased the latency to seizures (two-way ANOVA with repeated measures). **(D)** Statistical results showing AGM decreased the seizure duration (two-way ANOVA with repeated measures). Error bars represent mean ± standard deviation (n = 10 per group).#*p* ＜0.05 vs the control and PTZ group, *p＜0.05 vs the PTZ group and PTZ + AGM group.

The effect of agmatine on epileptic seizure susceptibility was investigated. Seizure stage score (stage 4 or higher), latency to seizure, incidence of seizure and seizure duration were used to evaluate seizure susceptibility in animals. We found that the mean seizure stage score in the PTZ group reached 4.60 ± 0.52, while the application of agmatine reduced the mean seizure stage score to 2.60 ± 0.52 (Figure 1A) (ANOVA, F (2,27) = 783.9 *p*＜0.05). Moreover, agmatine decreased the incidence of full kindling from 76.67 to 31.67% in PTZ-treated mice (Figure 1B). Agmatine also increased the latency to generalized seizures (ANOVA, F (1,18) = 7.484, *p*＜0.05) and reduced the duration of generalized seizures (ANOVA, F (1,18) = 102.4, *p*＜0.05) (Figures 1C,D). Moreover, we found that agmatine appears to be effective after 14 days. These results indicated that agmatine could alleviate seizure susceptibility in PTZ-treated mice.

### Agmatine Blocks Hippocampal Neuronal Damage in PTZ-Induced Mice

**FIGURE 2:**
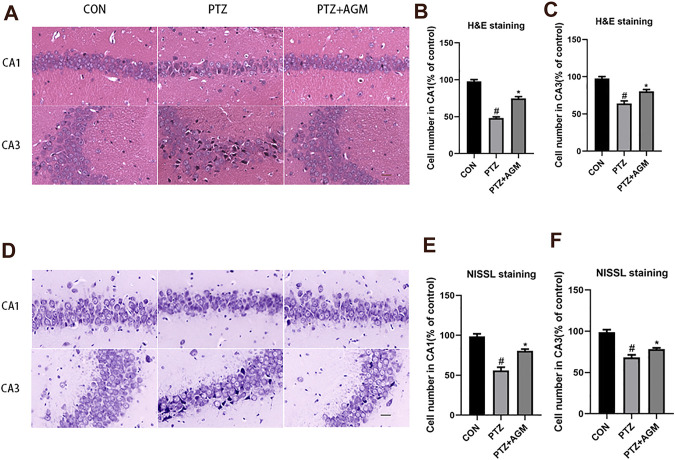
Effects of AGM on hippocampal neurol damage induced by PTZ kindling. **(A,D)** Representative micrographs (original magnification, ×200) showing AGM blocked hippocampal neuronal damage induced by PTZ kindling in CA1 and CA3 areas by HE staining and NISSL staining. Scale bar = 100px. **(B,C,E,F)** Statistical results showing AGM increase surviving cell number in CA1 and CA3 (% of control), (n = 4 mice per group, Student’s test). All data are shown as mean ± standard deviation.#*p* ＜0.05 vs the control and PTZ group, *p＜0.05 vs the PTZ group and PTZ + AGM group.

We also investigated the effects of agmatine on hippocampal neuronal damage induced by PTZ-induced kindling. Histological examination was performed by H&E and Nissl staining. As shown in Figure 2, hippocampal CA1 and CA3 areas of mice in the PTZ group showed serious damage compared with those of the control group. Agmatine alleviated hippocampal neuronal damage caused by PTZ-induced kindling. As indicated by Figures B–C, a dramatic increase of the cell number was observed in CA1 and CA3 regions of the hippocampus treated with agmatine compared to the PTZ-induced kindling mice (CA1: 74.75 2.63% versus 48.50 1.29%, *p*＜0.05; CA3: 80.50 2.08% versus 63.50 3.11%, *p*＜0.05). Also, as shown by Figure E–F, a significant increase in neuronal survival rate was noted in CA1 and CA3 regions in the hippocampus treated with agmatine compared to the PTZ-induced kindling mice (CA1: 80.67 2.08% versus 56.00 4.00%, *p*＜0.05; CA3: 78.33 1.53% versus 68.33 3.06%, *p*＜0.05). According to the result, the effect of agmatine alleviated hippocampal neuronal damage was more obvious in CA1 regions.

### GSDMD-N Levels Were Upregulated in the PTZ -Induced Mice and Agmatine Inhibits GSDMD-N Expression

**FIGURE 3:**
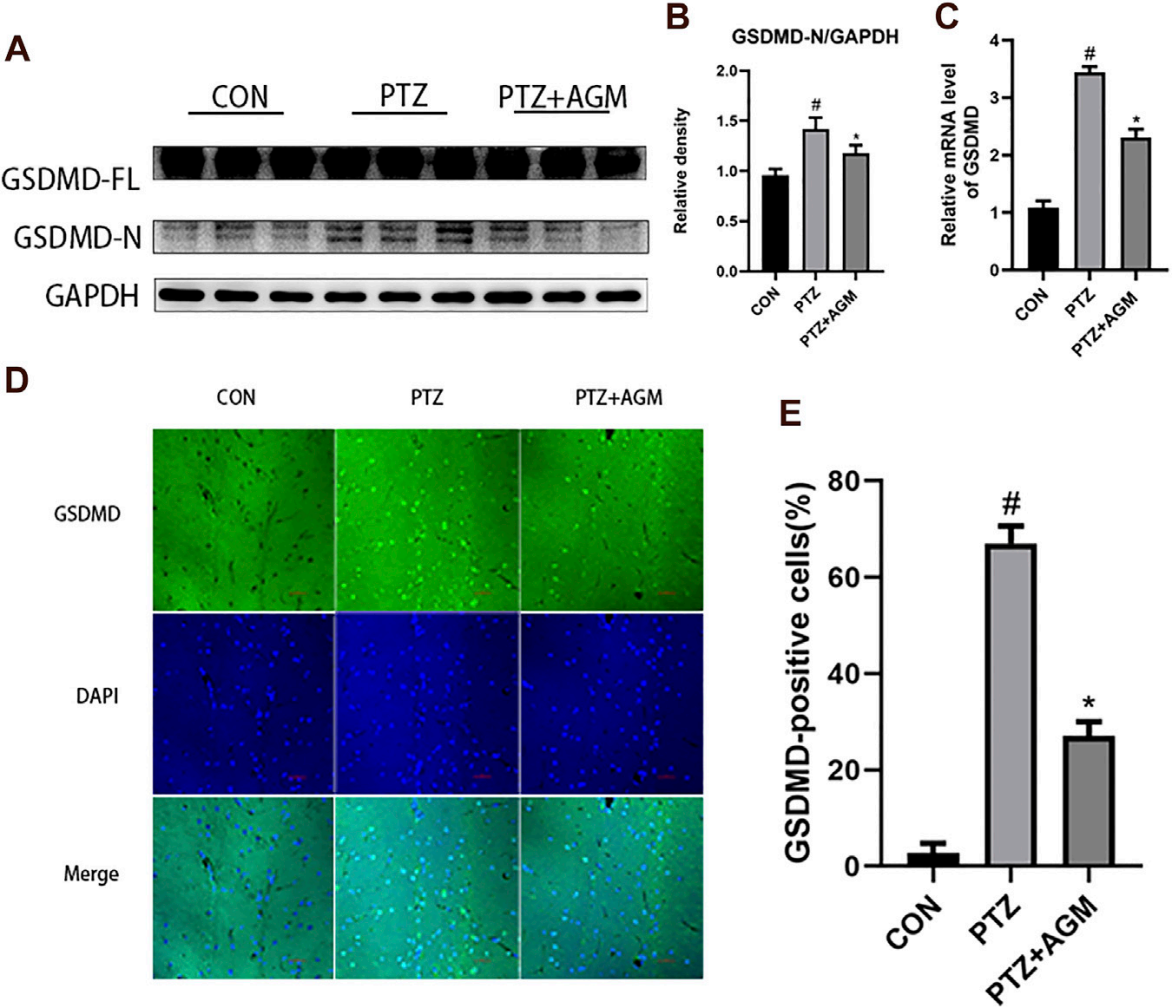
Effects of AGM on hippocampal GSDMD protein and mRNA in PTZ-induced kindling mice. **(A–B)** Proteins expression level of GSDMD-FL and GSDMD-N in different groups (n = 4 mice per group, Student’s test). **(C)** mRNA expression level of GSDMD in different group (n = 4 mice per group, Student’s test). **(D)** Representative imagines (original magnification,×400) showing AGM inhibited hippocampal neuronal GSDMD proteins expression induced by PTZ kindling. Scale bar = 100px. **(E)** Semi-quantitative analysis of the GSDMD-positive cells (%). The percentage of GSDMD-positive cells were defined as follows: 100 (count of GSDMD-positive cells/total count of cells), (*n* = 4 mice per group, Student’s test). All data are shown as mean ± standard deviation.#*p* ＜0.05 vs the control and PTZ group, *p＜0.05 vs the PTZ group and PTZ + AGM group.

The expression of GSDMD-N, the critical pyroptosis protein, was significantly increased at the protein and mRNA levels in the PTZ group compared with the control group (Figures 3A–C). However, the application of agmatine decreased the expression of GSDMD-N at the protein and mRNA levels. These results are consistent with the immunofluorescence staining results (Figures 3D,E). These data indicated that agmatine attenuated hippocampal neuronal damage and pyroptosis induced by PTZ kindling.

### Agmatine Inhibits NLRP3 Inflammasome and Inflammatory Cytokine Activation in PTZ-Kindled Mice and LPS-Induced Microglial Cells

**FIGURE 4:**
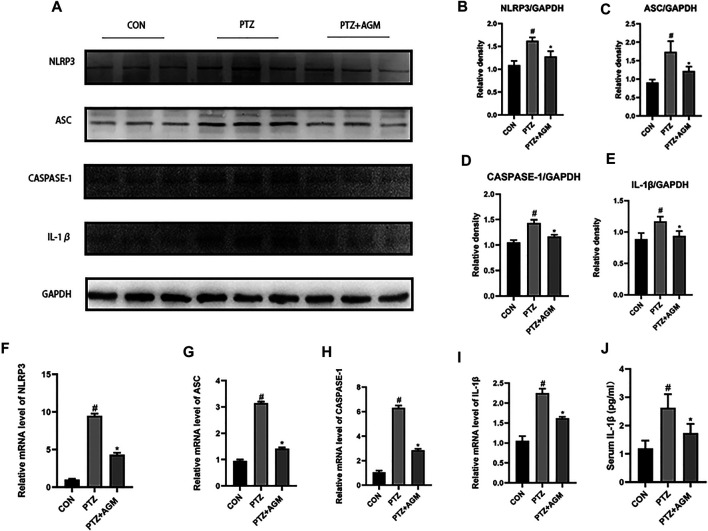
Effects of AGM on hippocampal inflammation-related proteins and mRNA in PTZ kindled mice. **(A–E)** Proteins expression level of inflammasome (NLRP3. ASC,CASPASE-1,IL-1β) indifferent groups (n = 4 mice per group, Student’s test). **(F–I)** mRNA expression level of inflammasome (NLRP3,ASC,CASPASE-1,IL-1β) in different groups (n = 4 mice per group, Student’s test). **(J)** The presence of IL-1β was measured by ELISA (n = 4 mice per group, Student’s test). All data are shown as mean ± standard deviation.#*p* ＜0.05 vs the control and PTZ group, *p＜0.05 vs the PTZ group and PTZ + AGM group.

The NLRP3 inflammasome is associated with neuroinflammation and epilepsy. To determine the effect of PTZ-induced kindling on the activation of the NLRP3 inflammasome, we examined the NLRP3 inflammasome in the hippocampus at the protein and mRNA levels. As shown in Figure 4, the protein and mRNA expression of NLRP3 in mice in the PTZ group was significantly increased compared with that of mice in the control group, while agmatine inhibited the expression of NLRP3. Similarly, agmatine also attenuated the increase in ASC, Caspase-1 and IL-1β expression induced by PTZ kindling, and we also found that the level of IL-1β in the hippocampus was decreased, as detected by ELISA, which was consistent with the western blot results.

**FIGURE 5:**
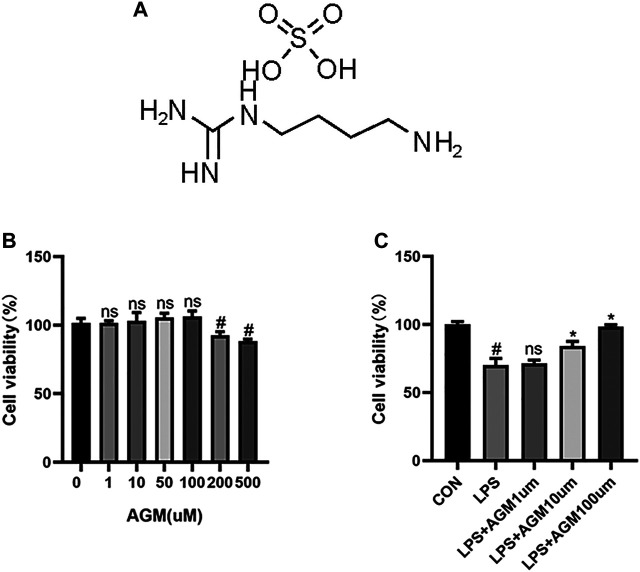
CCK8 analysis of the effect of AGM on viability of BV2 microglial cells. **(A)** Structure of AGM. **(B)** Viability of BV2 microglial cells treated with AGM in graded concentrations (0,1,10,50,100,200,500) was measured by CCK8 assay (one-way ANOVA followed by Dunnett’s test). **(C)** Viability of BV2 microglial cells treated with AGM in graded concentrations (1,10,100) was measured by CCK8 assay (one-way ANOVA followed by Dunnett’s test). All data are shown as mean ± standard deviation.#*p* ＜0.05 vs the control and LPS group, *p＜0.05 vs the LPS group and LPS + AGM group.

The activation of microglia could exacerbate neuroinflammation and cause or exacerbate seizures and we evaluated the neuroprotective effects of agmatine by using BV2 microglial cells (Ahn et al., 2012). We used 0–500 μM agmatine to find the best concentration. As shown in Figure 5, agmatine at 200 and 500 μM decresed cell viability. Therefore, we chose agmatine at concentrations of 1,10, and 100 μM for the next experiment (Figure 5C), and found that 100 μM agmatine was the optimal concentration. Therefore, 100 μM agmatine was used in the subsequent experiments.

**FIGURE 6:**
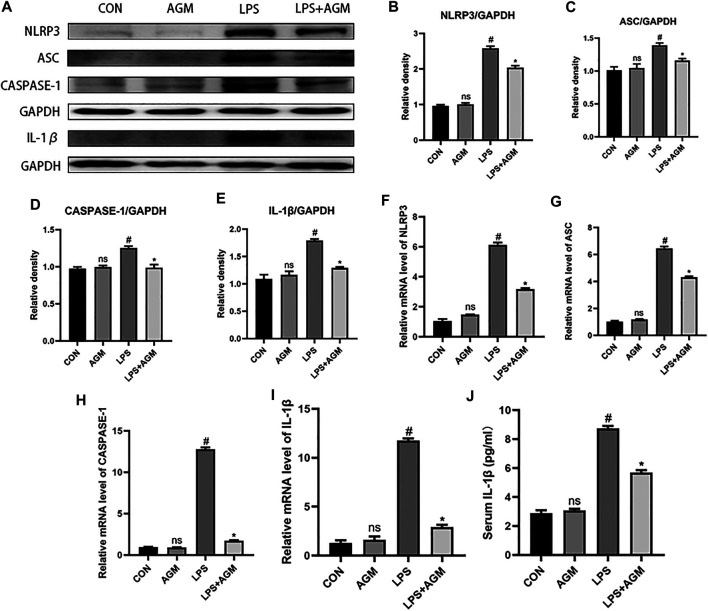
Effects of AGM on inflammation-related proteins and mRNA in LPS-induced BV2 microglial cells. **(A–E)** Proteins expression level of inflammasome (NLRP3. ASC,CASPASE-1,IL-1β) in different groups (Student’s test). **(F–I)** mRNA expression level of inflammasome (NLRP3,ASC,CASPASE-1,IL-1β) in different groups (Student’s test). **(J)** The presence of IL-1β was measured by ELISA (Student’s test). All data are shown as mean ± standard deviation.#*p* ＜0.05 vs the control and LPS group, *p＜0.05 vs the LPS group and LPS + AGM group.

LPS activated the NLRP3 inflammasome in BV2 microglia, and significantly increased the protein and mRNA expression of NLRP3, ASC, Caspase-1 and IL-1β. Agmatine significantly reduced the protein and mRNA expression of NLRP3, ASC, Caspase-1 and IL-1β. In addition, the ELISA results also demonstrated that the cytokine IL-1β was significantly higher in the LPS group than in the control group. However, agmatine significantly inhibited LPS-induced IL-1β expression. Interestingly, we observed that the application of agmatine alone did not affect NLRP3 inflammasome activation (Figure 6). These data indicated that agmatine was involved in the inhibition of NLRP3 inflammasome-mediated inflammatory processes in PTZ-kindled mice and in LPS-induced BV2 microglial cells.

### Inhibition of Pyroptosis by Agmatine Involves the TLR4/MYD88/NF-κB/NLRP3 Inflammasome Pathway

**FIGURE 7:**
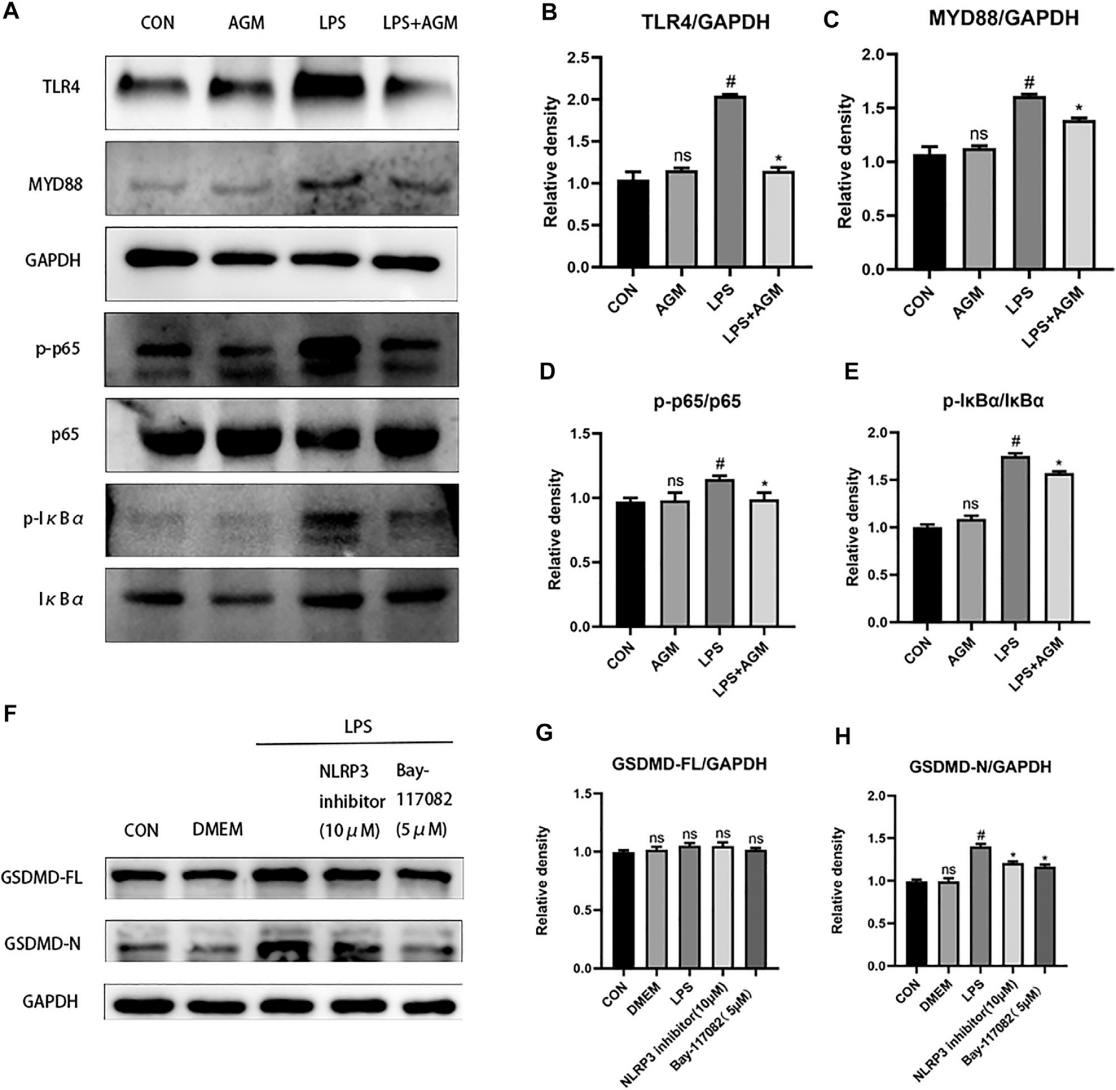
AGM inhibited the TLR4/MyD88/NF-κB/NLRP3 inflammasome pathway in LPS-induced BV2 microglial cells. **(A–E)** Proteins expression level of inflammasome pathway (TLR4, MYD88, p-p65, p65, *p*-IκBα, IκBα) in different groups (Student’s test). **(F–H)** Proteins expression level of GSDMD-FL and GSDMD-N in different groups (Student’s test). All data are shown as mean ± standard deviation.#*p* ＜0.05 vs the control and LPS group, *p＜0.05 vs the LPS group and LPS + AGM group. *p＜0.05 vs the LPS group and NLRP3 inhibitor (10 μM), Bay-117082 (5 μM).

**FIGURE 8:**
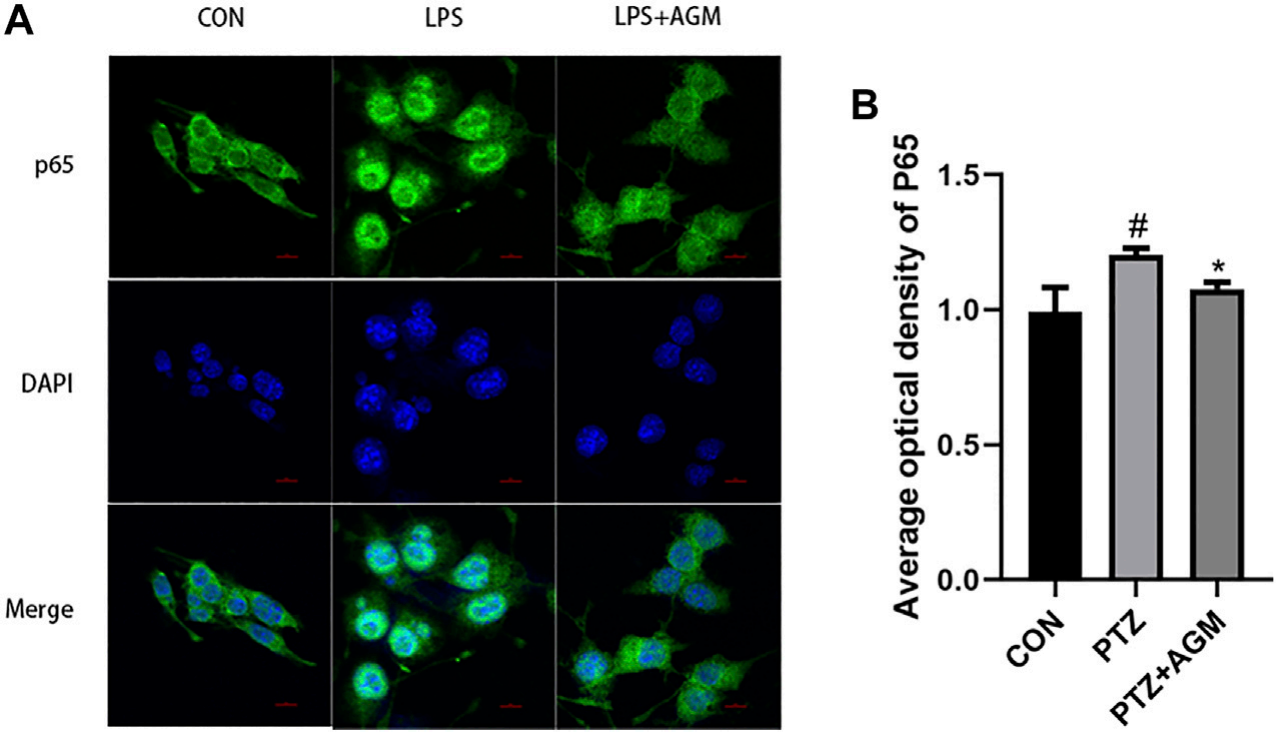
Effects of AGM on the expression of P65 activated by LPS-induced BV2 microglial cells. **(A)** Representative images (original magnification,×400) of immunofluorescence staining of P65 in BV2 microglial cells. Scale bar = 100px. **(B)** Semi-quantitative analysis of the relative levels of P65 by densitometric analysis (Student’s test). #*p* ＜0.05 vs the control and LPS group, *p＜0.05 vs the LPS group and LPS + AGM group.

TLR4/MYD88/NF-κB was associated with the activation of the NLRP3 inflammasome. We observed that the expression of TLR4 was increased in LPS-induced BV2 microglial cells, while agmatine inhibited the expression of TLR4 (Figure 7). TLRs activate the intracellular TLR-domain-containing adaptor protein MYD88, triggering downstream activation of NF-κB. We found increased protein expression of MYD88, *p*-IκBα, p-p65, ASC and NLRP3 in LPS-induced BV2 microglial cells. Agmatine inhibited the expression of MYD88, p- IκBα, p-p65, ASC and NLRP3. Cell morphology was observed by immunostaining with p65 antibodies (Figure 8). Moreover, LPS-stimulated BV2 microglial cells were significantly different compared with control group cells, which were similar to agmatine-treated BV2 microglia. Studies have shown that the NF-κB/NLRP3 inflammasome is involved in the activation of Caspase-1 (Man and Kanneganti, 2015), and we further explored whether inhibiting the NF-κB/NLRP3 inflammasome could reduce pyroptosis. Before LPS stimulation of BV2 microglia, we treated the cells with 10 μM NLRP3 inflammasome inhibitor or 5 μM Bay-117082 (an NF-κB pathway inhibitor) for 1 h. We found that the expression of GSDMD-N was inhibited when the cells were cultured with the NLRP3 inflammasome inhibitor or Bay-117082. These results suggest that agmatine is involved in the inhibition of the TLR4/MyD88/NF-κB inflammasome pathway in BV2 microglia.

## Discussion

The present study revealed that agmatine inhibited chronic seizures in mice with PTZ-induced kindling. We observed that agmatine could delay the latency of PTZ kindling, reduce the severity of seizures and attenuate hippocampal neuronal damage. Moreover, the anti-inflammatory effects of agmatine may be related to activation of the TLR4/MYD88/NF-κB/NLRP3 pathway *in vitro*. Furthermore, agmatine has been shown to block the activation of the core pyroptosis protein GSDMD and decrease the expression of the inflammasome. Therefore, these findings suggest that agmatine could affect epileptogenesis, alleviate seizures and play a neuroprotective role, which may be due to the inhibition of pyroptosis and neuroinflammatory responses.

Epilepsy is a neurological disease with a complex aetiology. Imbalances in excitement and inhibition may be the main cause of epilepsy (Scharfman, 2007). Some studies have shown that the occurrence and development of epilepsy are closely related to inflammation (Mao et al., 2017), which is considered to be a biomarker of epileptogenesis (Vezzani and Friedman, 2011). The inflammatory response is a balance between pro-inflammatory cytokines such as IL-1β, and anti-inflammatory cytokines such as IL-10 (Adib-Conquy and Cavaillon, 2012). IL-1β stimulates neurons to express IL-1 receptor type 1 and active src protein kinase, mediate the hyperphosphorylation of N-methyl-D-aspartic acid (NMDA) receptor NR2B subunits, increase calcium influx and mediate hyperexcitability and excitotoxicity in neurons (Balosso et al., 2008), thus increasing the risk of seizures (Gao et al., 2018).

Agmatine, an NMDA receptor inhibitor, has been reported to alleviate chronic seizures (Xu et al., 2014). A chronic epilepsy model induced by PTZ kindling was established in this study. We found that agmatine could decrease the severity and incidence of fully kindled seizures. It is well-known that the hippocampus is critical for learning in mammals (Bohbot and Corkin, 2007). Previous studies have shown that PTZ-kindled seizures lead to hippocampal neuronal damage (Kaur et al., 2015). Our results suggest that agmatine blocks neuronal damage in the hippocampal CA1 and CA3 areas of PTZ-kindled mice, which is consistent with the findings of our previous studies (Xu et al., 2014).

Studies have shown that as a new type of pro-inflammatory programmed cell death, pyroptosis can occur in acute liver injury (Chen et al., 2016), diabetic cardiomyopathy (X. Li et al., 2014), Alzheimer’s disease (M. S. Tan et al., 2014), traumatic brain injury (Adamczak et al., 2014), ethanol-induced brain injury (Alfonso-Loeches et al., 2014) and myocardial ischaemia/reperfusion (Qiu et al., 2017). Moreover, pyroptosis is also closely related to epilepsy (Tan et al., 2015), however, it is unknown whether the core pyroptosis protein GSDMD is associated with the pathogenesis of epilepsy. The present study demonstrated that epileptic seizures activated GSDMD and increased GSDMD-N levels through PTZ kindling and that agmatine reduced GSDMD activation, thereby alleviating seizures. It has been reported that GSDMD is activated by the NLRP3 inflammasome, which then cleaves GSDMD to produce GSDMD-N, and GSDMD-N is translocated to the plasma membrane to form a pore; then, the cell membrane swells and ruptures, resulting in the release of cytoplasmic contents and the leakage of mature IL-1β and IL-18 (Ding et al., 2016). Studies have shown that the levels of IL-1β are elevated in dogs with epilepsy (Kostic et al., 2019) and in hippocampal sclerosis patients with mesial temporal lobe epilepsy (Dundar et al., 2013). We found that agmatine could reduce the expression of NLRP3, ASC, caspase-1 and IL-1β at the protein and mRNA levels, and inhibit the release of IL-1β in serum and supernatant, thus inhibiting neuroinflammation, GSDMD activation, and pyroptosis.

**FIGURE 9:**
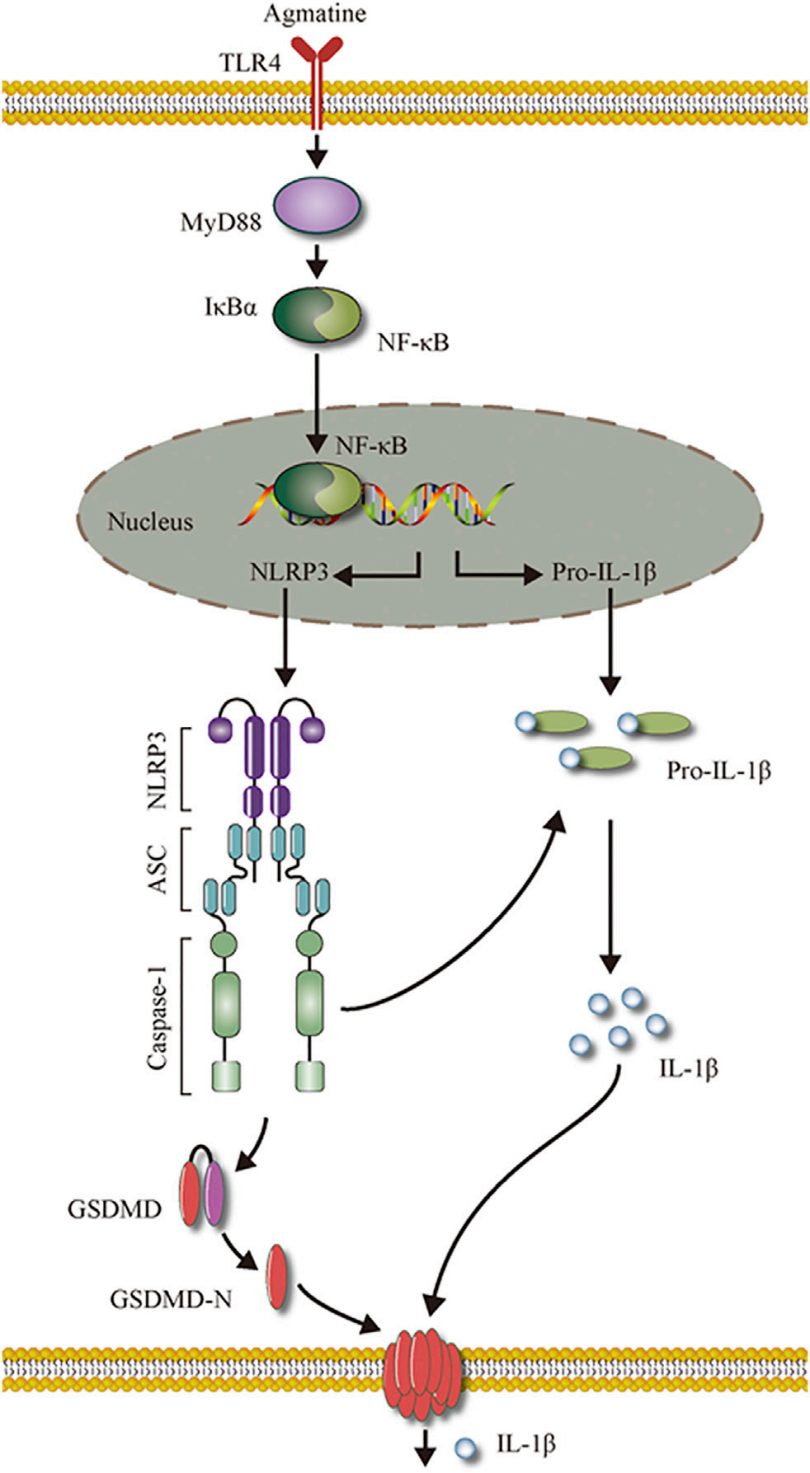
The schematic model of the role of agmatine on pyroptosis in seizures.

TLRs are a family of pattern recognition receptors, most of which are expressed in the central nervous system (Takeda et al., 2003). TLR4 is the first-characterized mammalian TLR and plays a crucial role in various neuropathological processes, including neuroinflammation and neuronal injury (Rahimifard et al., 2017). Some studies have shown that intrahippocampal application of a TLR4 antagonist could reduce seizure susceptibility in mice (Maroso et al., 2010) and exert neuroprotective and anti-inflammatory effects on a seizure model (Li et al., 2013), indicating that inflammation is an important therapeutic target to improve the prognosis of epilepsy. TLR4 has been shown to be expressed in microglial cells (Zhao et al., 2016). Microglial cells are closely related to epilepsy. Epilepsy can stimulate the activation of microglial cells (Morin-Brureau et al., 2018), worsen inflammation in the brain and exacerbate seizures (Hiragi et al., 2018). Although TLR4 plays an important role in sepsis (Li et al., 2020), it can also be activated by LPS(Zhang et al., 2020). TLR4 recruits MYD88, activates NF-κB, and increases the synthesis of NLRP3 and pro-IL-1β(Afonina et al., 2017; Ve et al., 2017). Some studies have shown that melatonin can reduce inflammation-induced pyroptosis in mouse adipose tissue by blocking NF-κB/GSDMD signalling (Liu et al., 2017). In our study, we examined whether NF-κB and the NLRP3 inflammasome were involved in LPS-induced pyroptosis. We found that NF-κB or the NLRP3 inflammasome inhibitor decreased the expression of GSDMD-N. Moreover, agmatine reduced the expression of TLR4, MYD88, NF-κB and the NLRP3 inflammasome in microglial cells, and reduced inflammation in the brain, thereby reducing seizures and protecting neurons (Figure 9). Although experimental animal and cell models have been used for elucidating that agmatine alleviates epileptic seizures and hippocampal neuronal damage by inhibiting gasdermin D-mediated pyroptosis, further experimental research and rigorous clinical investigation should be conducted via human studies.

## Conclusion

The present study demonstrates that PTZ-induced kindling increases the susceptibility of mice to seizures and pyroptosis, and enhances the expression of GSDMD, NLRP3 inflammasome and IL-1β inflammatory cytokines *in vivo* and *in vitro*. The application of agmatine can reduce these effects. The inhibitory effect of agmatine on pyroptosis is mediated by suppressing the TLR4/MYD88/NF-κB/NLRP3 inflammasome pathway. Moreover, the TLR4/MYD88/NF-κB/NLRP3 inflammasome pathway may be an important target for epilepsy treatment. Thus, agmatine may be a potential drug for the treatment of seizures.

## Data Availability

The original contributions presented in the study are included in the article/Supplementary Material, further inquiries can be directed to the corresponding author.

## Abbreviations

PTZ, pentylenetetrazole; GSDMD, gasdermin D; GSDMD-N, gasdermin D-N domains; IL-1β, inflammatory cytokines Interleukin-1β; LPS, lipopolysaccharide; AGM, agmatine; MJs, myoclonic jerks; GTCSs, generalized tonic-clonic seizures; DMEM, Dulbecco’s modified Eagle’s medium; FBS, fetal bovine serum; NLRP3, Nucleotide oligomerization domain (NOD)-like receptor protein 3; ASC, apoptosis-associated speck-like protein containing a caspase-activating recruitment domain; CCK8, cell counting kit 8; WST-8, water-soluble tetrazolium salt; ELISA, enzyme-linked immunosorbent assay; H&E, Haematoxylin and eosin; NMDA, N-methyl-D-aspartic acid.
