# Supplementary figures and images for: Agmatine Alleviates Epileptic Seizures and Hippocampal Neuronal Damage by Inhibiting Gasdermin D-Mediated Pyroptosis
Source: Front Pharmacol. 2021 Aug 6;12:627557. doi: 10.3389/fphar.2021.627557 (PMC8378273; doi:10.3389/fphar.2021.627557)

Figure4:

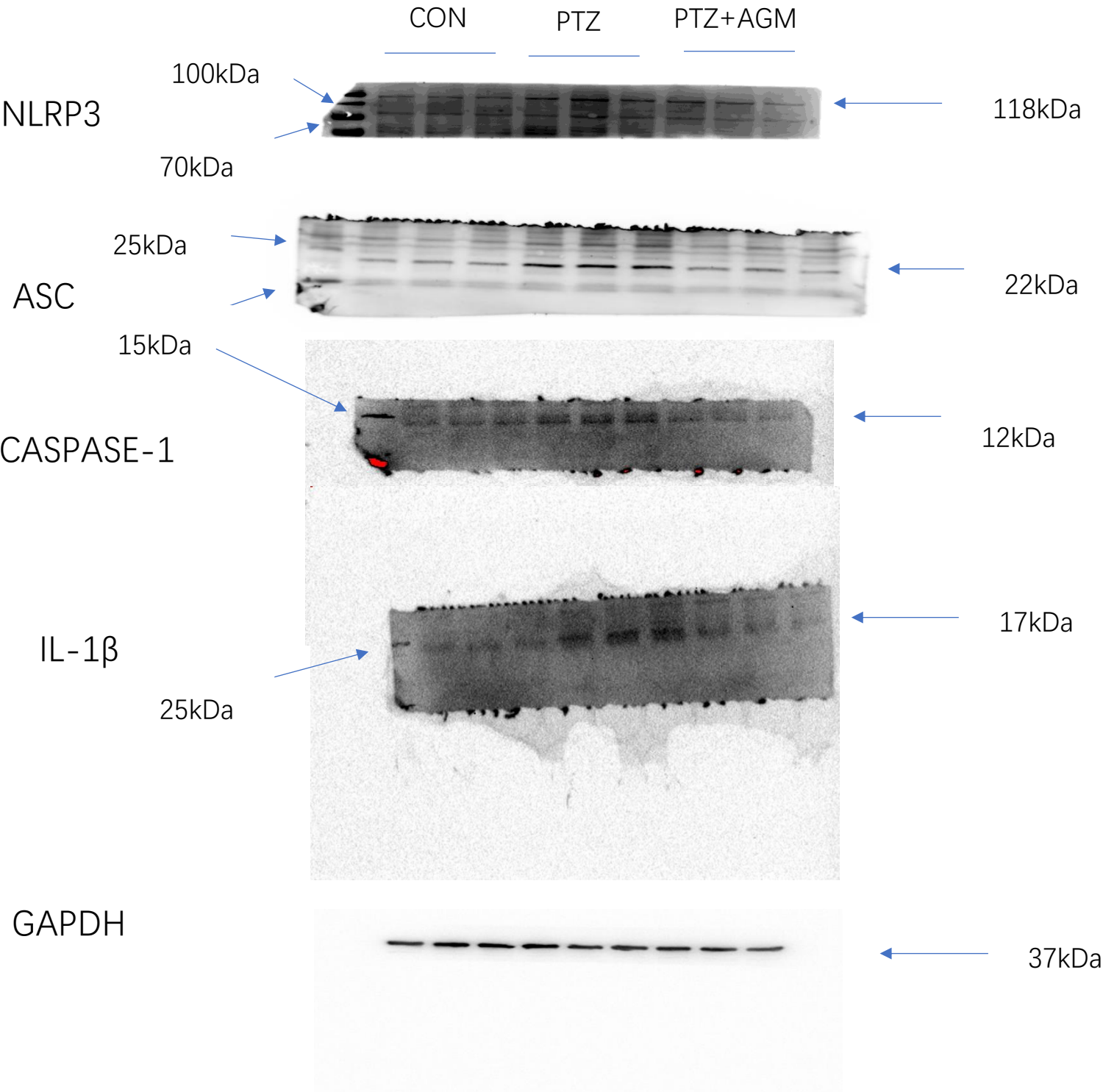

Supplement: Supplementary file 1 [file DataSheet2.PDF]

Figure7:

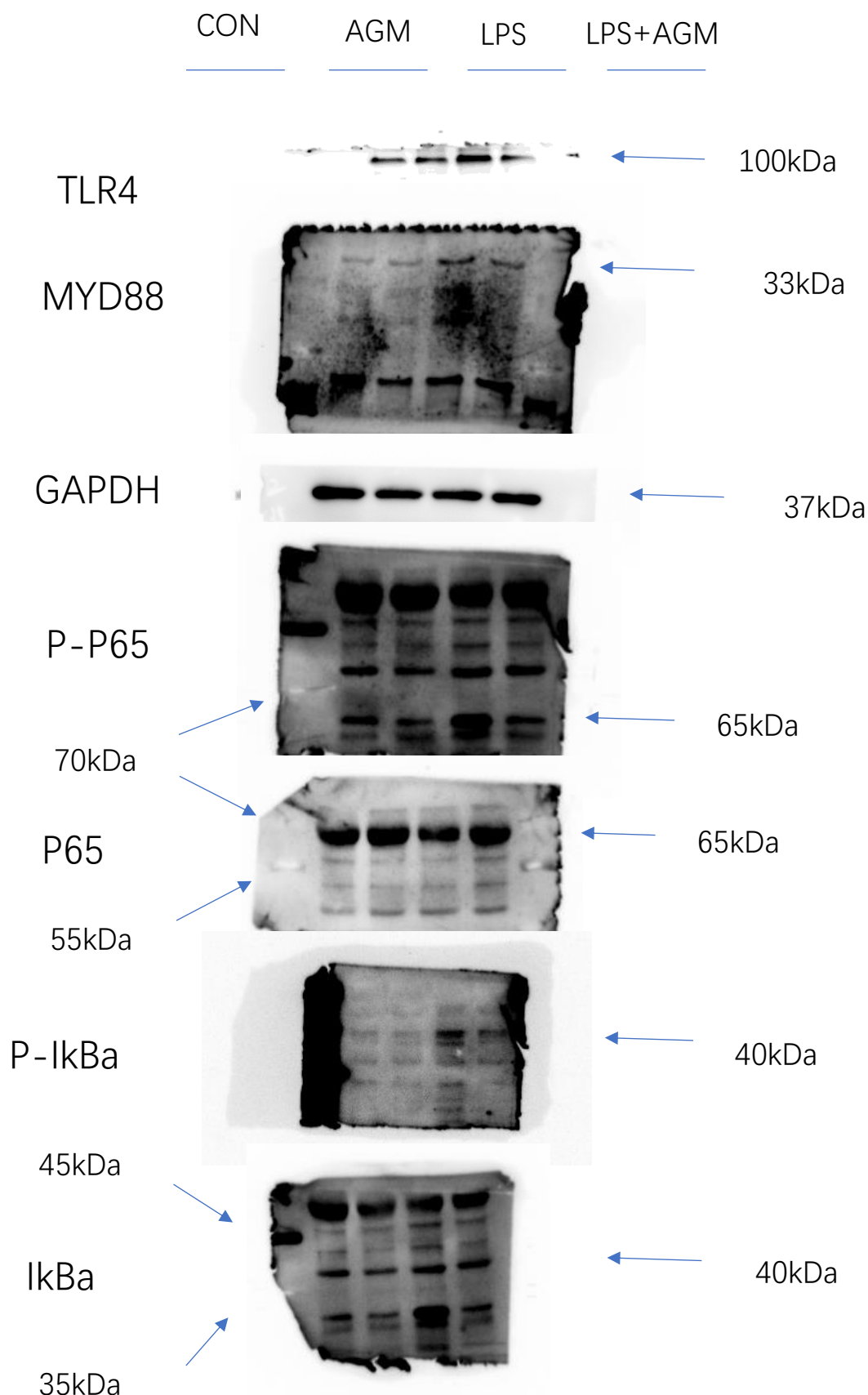

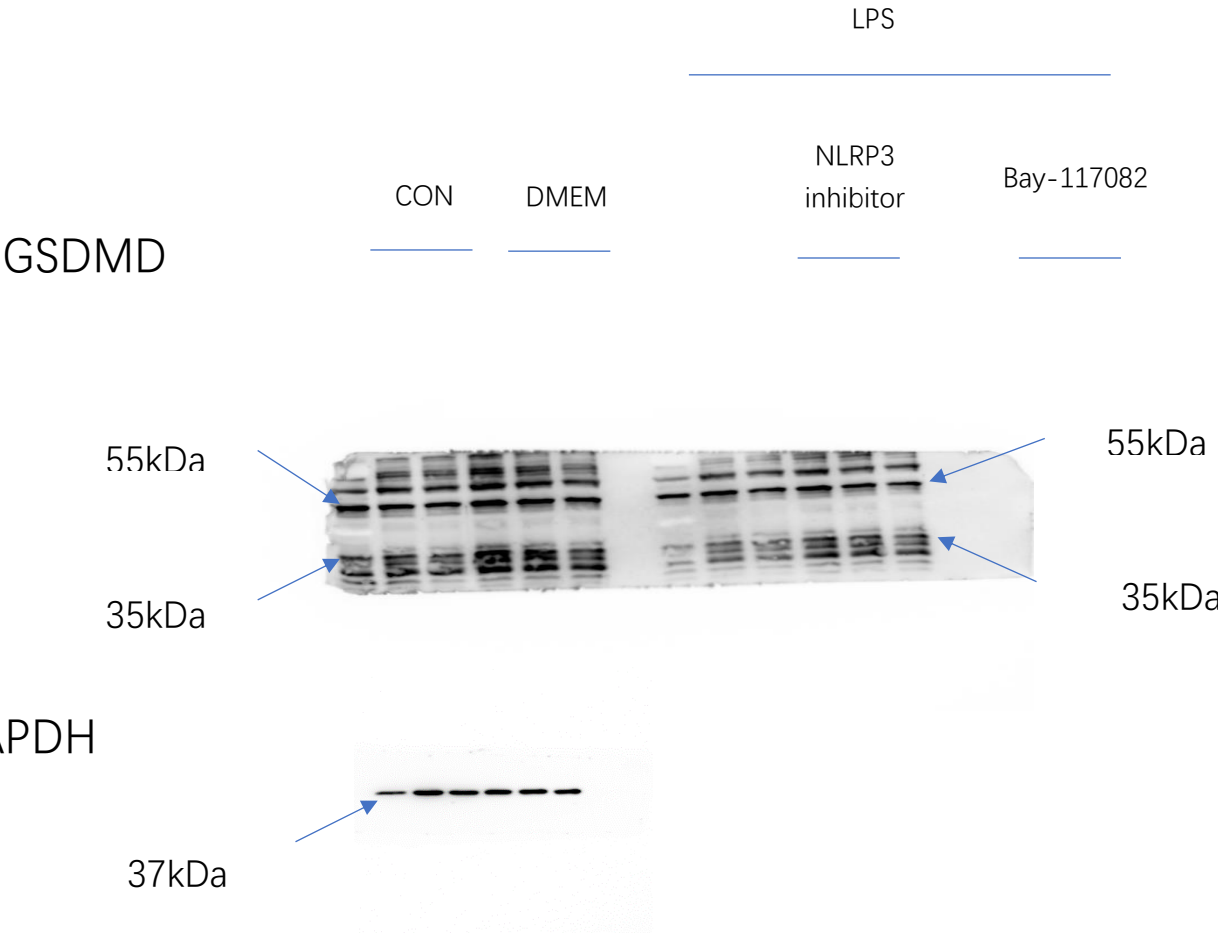

Supplement: Supplementary file 2 [file DataSheet4.PDF]

Figure6:

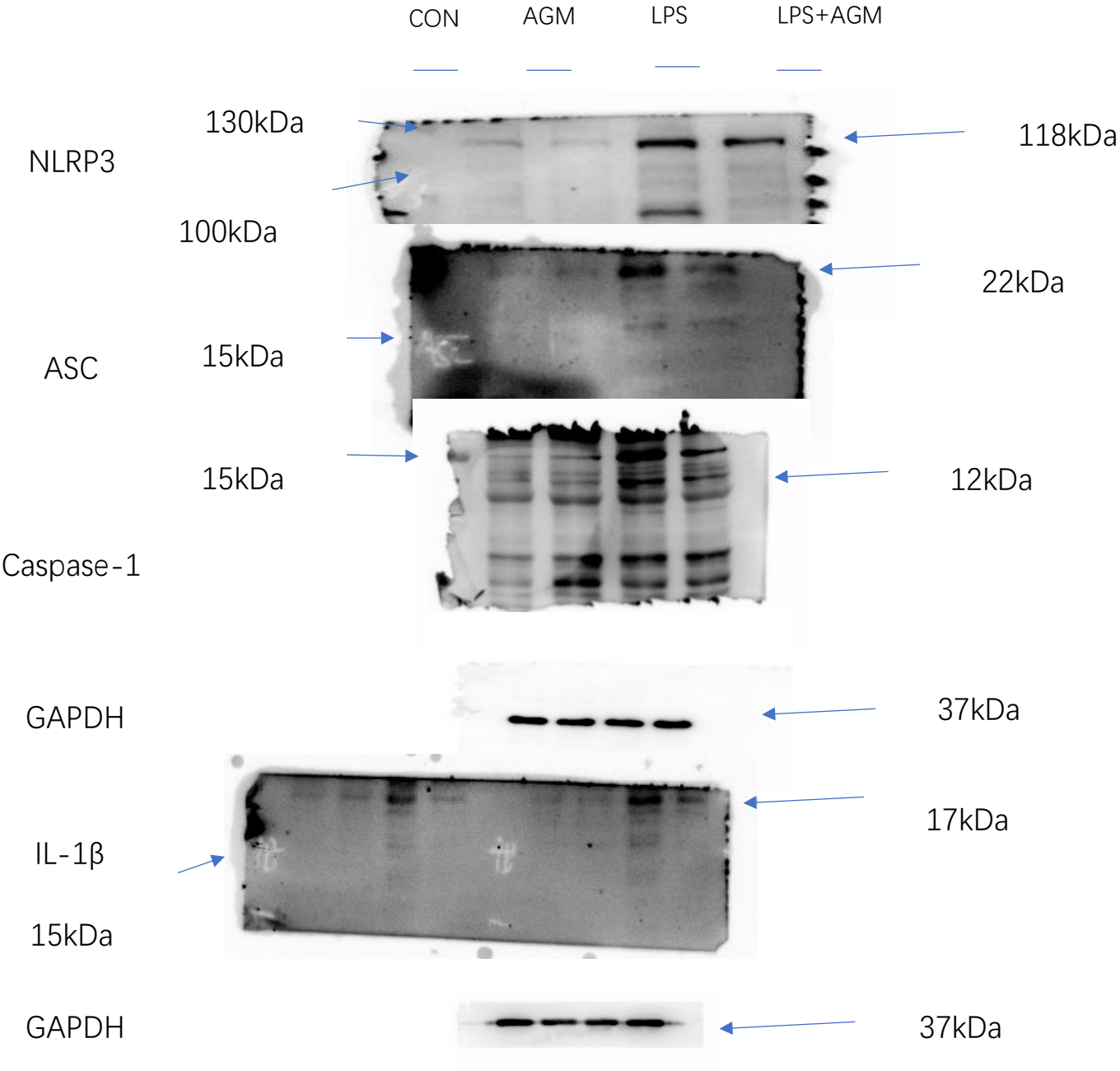

Supplement: Supplementary file 3 [file DataSheet3.PDF]

Figure3:

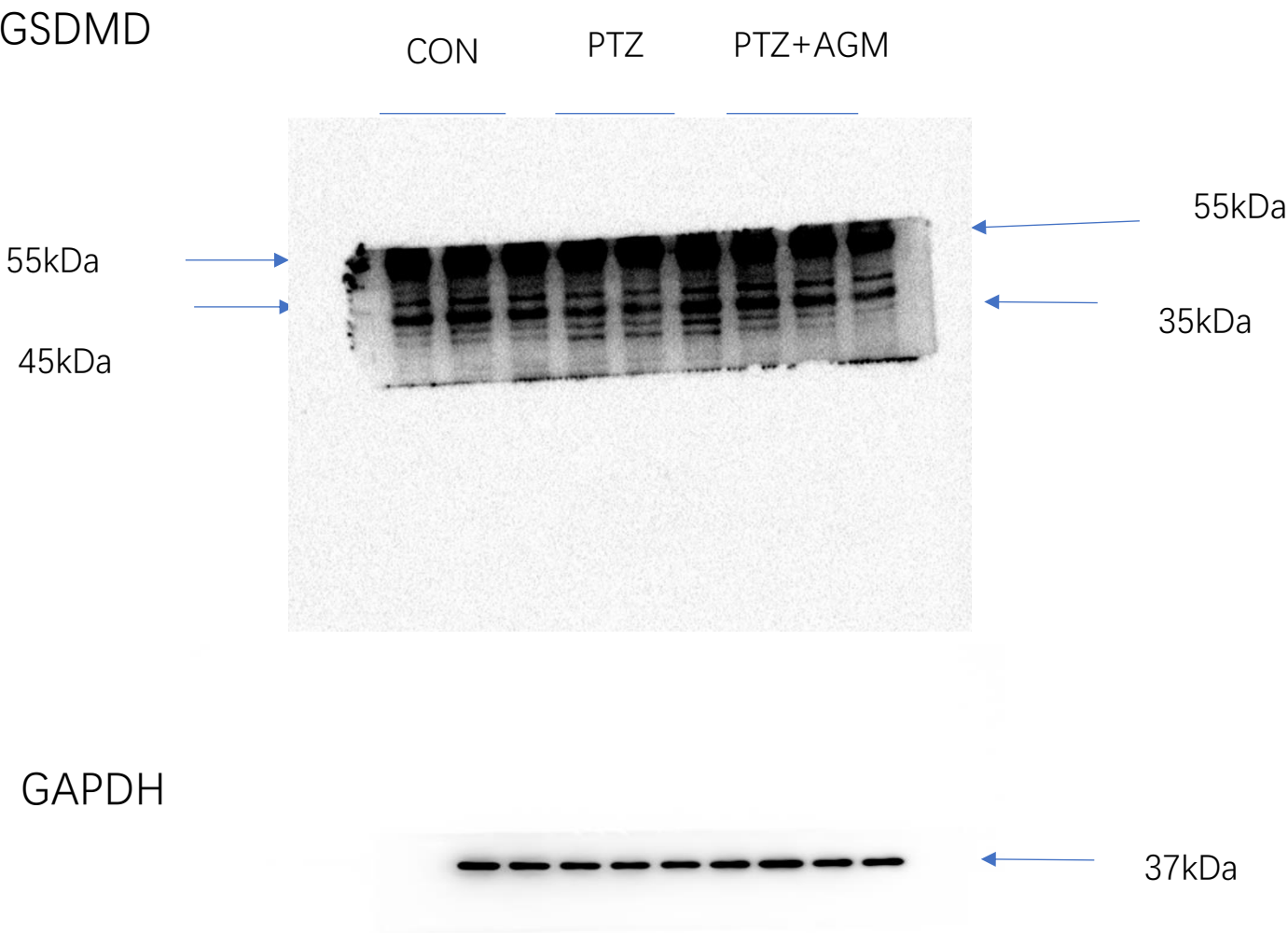

Supplement: Supplementary file 4 [file DataSheet1.PDF]
